# Low level activity thresholds for changes in NMR biomarkers and genes in high risk subjects for Type 2 Diabetes

**DOI:** 10.1038/s41598-017-09753-6

**Published:** 2017-09-18

**Authors:** Karl-Heinz Herzig, Juhani Leppäluoto, Jari Jokelainen, Emmanuelle Meugnier, Sandra Pesenti, Harri Selänne, Kari A. Mäkelä, Riikka Ahola, Timo Jämsä, Hubert Vidal, Sirkka Keinänen-Kiukaanniemi

**Affiliations:** 1 Research Unit of Biomedicine, and Biocenter of Oulu, Oulu University, 90014 Oulu, Finland; 2 Department of Gastroenterology and Metabolism, Poznan University of Medical Sciences, Poznan, Poland; 3 Medical Research Center and Oulu University Hospital, University of Oulu and Oulu University Hospital, Oulu, Finland; 4 Center for Life Course Health Research, Faculty of Medicine, University of Oulu, 90014 Oulu, Finland; 5 Oulu University Hospital, Unit of General Practice, and Health Center of Oulu, Oulu, Finland; 6 CarMeN Laboratory, INSERM U1060, INRA U1397, University of Lyon, 69600 Oullins, France; 7 Department of Education and Psychology, University of Jyväskylä, Jyväskylä, Finland; 8 Research Unit of Medical Imaging, Physics and Technology, University of Oulu, 90014 Oulu, Finland; 9 Department of Diagnostic Imaging, Oulu University Hospital, Oulu, Finland

## Abstract

Our objectives were to determine if there are quantitative associations between amounts and intensities of physical activities (PA) on NMR biomarkers and changes in skeletal muscle gene expressions in subjects with high risk for type 2 diabetes (T2D) performing a 3-month PA intervention. We found that PA was associated with beneficial biomarker changes in a factor containing several VLDL and HDL subclasses and lipids in principal component analysis (P = <0.01). Division of PA into quartiles demonstrated significant changes in NMR biomarkers in the 2nd - 4th quartiles compared to the 1st quartile representing PA of less than 2850 daily steps (P = 0.0036). Mediation analysis of PA-related reductions in lipoproteins showed that the effects of PA was 4–15 times greater than those of body weight or fat mass reductions. In a subset study in highly active subjects’ gene expressions of oxidative fiber markers, Apo D, and G0/G1 Switch Gene 2, controlling insulin signaling and glucose metabolism were significantly increased. Slow walking at speeds of 2–3 km/h exceeding 2895 steps/day attenuated several circulating lipoprotein lipids. The effects were mediated rather by PA than body weight or fat loss. Thus, lower thresholds for PA may exist for long term prevention of cardio-metabolic diseases in sedentary overweight subjects.

## Introduction

Physical activity has been decreasing due to our changes in life style and our environment while major diseases like diabetes, cardiovascular diseases, cancer, chronic respiratory diseases and mental disorders have been increasing accounting for the majority of the disease burden in Europe. In addition, the number of adults with type 2 diabetes (T2D) has near quadrupled worldwide especially in low-income and middle-income countries^1^. Salutary effects of improved life style factors on T2D progression in high risk subjects are well documented^2, 3^, but it is unknown how changes in physical activity and body weight mediate health effects in high risk subjects.

Effects of long-term exercises or high levels of physical activities on circulating metabolites have previously been studied in healthy subjects. Most of the VLDL, LDL and HDL particle concentrations, VLDL-triglycerides, LDL-cholesterol, apolipoprotein B, glycoprotein acetylated, and various fatty acids, amino acids and metabolites have been shown to decrease after exercises in physically active subjects^4–7^. Recently, the effects of objectively measured low whole-day physical activity on changes in conventionally measured glucose, insulin, cholesterol and lipids were investigated in sedentary and obese subjects with abnormal glucose tolerance during a 3 months intervention^8^. Basal, post-load insulin and HOMA-IR decreased when the number of daily steps exceeded 5500 at the acceleration levels of steps between 1.3–1.7 g. Furthermore, in the most active quartile (Q) of subjects’ triglycerides, total and LDL cholesterol and visceral fat were lower than in the least active one, when the number of daily steps exceeded 6500. Furthermore, population and prospective studies indicate that type 2 diabetes (T2D) is associated with elevated concentrations of several lipoprotein lipids^9–12^.

In this study we determined lipoprotein lipid subclasses, triglycerides and fatty acids, known to correlate with cardio-metabolic risks, and their associations with whole day physical activities in the participating subjects. In addition, in a subset of subjects, who were willing to undergo muscle biopsies, we performed gene expression profiling in the quadriceps muscle to evaluate changes in the transcription level of potential candidate genes related to fatty acid oxidation and glucose and lipid metabolism.

Our working hypothesis has been that beneficial effects of objectively quantified physical activity are associated with changes in one or more circulating NMR biomarkers and skeletal muscle gene expression. We speculated that that there might be physical activity thresholds for these measures that can be used for planning exercise programs for sedentary subjects with high T2D risks since it has been shown repeatedly that the high risk groups are unable to follow the recommended 150 min PA guideline^13, 14^.

## Results

**Figure 1:**
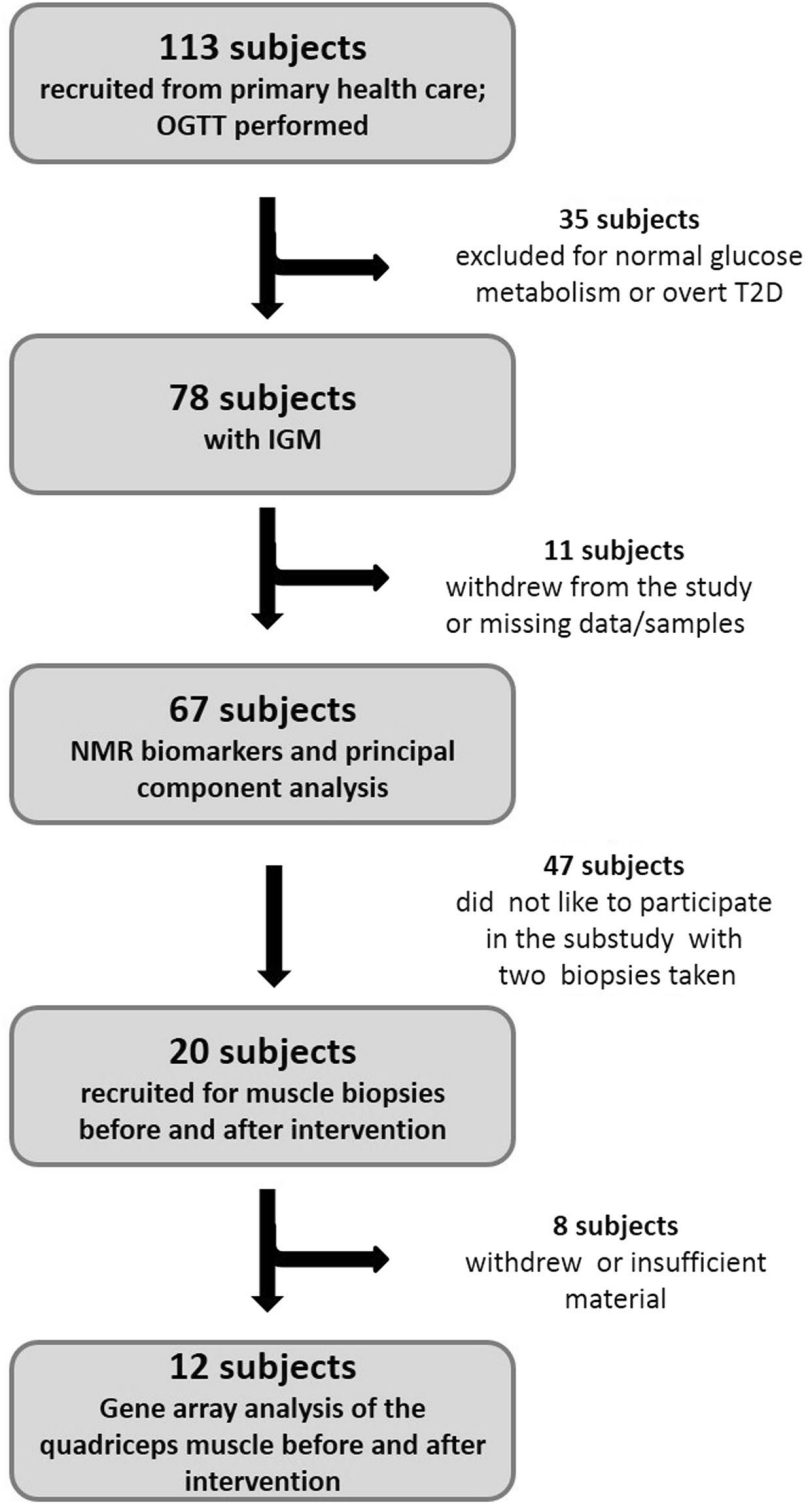
Flow chart of the study participants. OGTT – oral glucose tolerance test; IGM – impaired glucose tolerance.

Approximately 5 * 10^7^ steps and accelerations were recorded in the main study. Of the walking induced steps over 80% fell into the accelerations levels of 1.3–1.7 g, corresponding to the walking speed of 2–3 km/h^8^. The mean daily numbers of the steps were 5870 in the subjects participating to structured exercises and 4434 in the control subjects (p < 0.05). According to the best standard of care all participants (Fig. 1) were informed about the significance of regular exercise and weight reduction to prevent T2D during three common meetings. This resulted in a co-intervention effect with high PA levels and significant weight reductions in the control groups^8^. Therefore, the differences between the variables were analyzed in PA quartiles.

In the intervention group the mean body weight loss of the subjects was 1.1 kg with no significant difference between intervention and control subjects^8^. Skeletal muscle mass changed +0.8 kg in the intervention and −0.1 kg in the control group (both changes not significant). The nutritional intake was monitored by 3-day food questionnaires, yet no significant changes before and after the intervention were observed.

**Figure 2:**
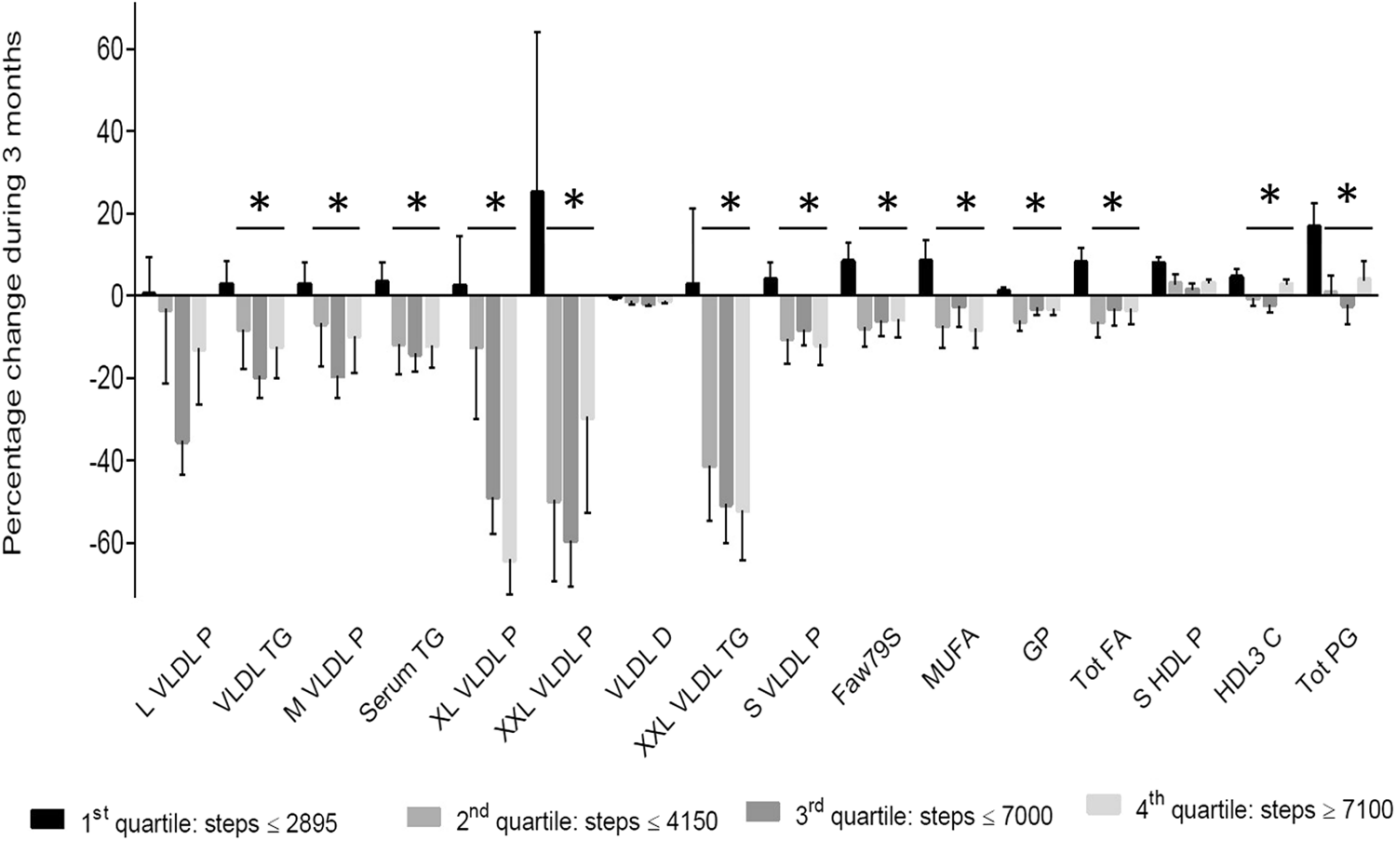
Percentage changes in lipoprotein lipids, triglycerides, fatty acids and glycoprotein in the physical activity quartiles (number of daily steps) and SDs (vertical bars). Horizontal lines with asterisks above the quartiles 2nd–4th indicate significant reductions (p < 0.0036) between the 1st and other quartiles. Data were log transformed and adjusted to baseline, sex and age. Numbers of observations were 17 in each quartile. For abbreviations P present particles, L VLDL – large VLDL, XL – Extra Large; XXL - Extremely Large, TG- triglycerides, D - diameter, Faw79S - ω7 and ω 9 fatty acids (FA), MU - monounsaturated, GP - glycoprotein acetyl, Tot - total, C - cholesterol and PG - phosphoglycerides.

The associations between daily steps or body weight changes and 11 factors from PCA were analyzed. Only factor 1 consisting of several VLDL particles, fatty acids and lipids showed significant inverse correlation (Table 1 and Supplementary Table 2). In order to quantify effects of physical activity on the changes in the factor 1 variables, the lowest active quartiles were compared with other quartiles. Changes in 13 out of 16 factor 1 variables (medium, very large, extremely large and small VLDL particles, triglycerides in VLDL, extremely large VLDL and serum, ω-7 and ω-9 fatty acids, monounsaturated fatty acids, total fatty acids, glycoprotein acetyl, HDL3 cholesterol and total phosphoglycerides) in the Q2–Q4 were significantly lower than those in the Q1. The reductions took place at as low as >2895 daily steps (Fig. 2). In addition, there were also significant positive correlations between body weight change and factor 1 variables as well as with basal insulin, post load glucose and HOMA (factor 7: LDL particle size, medium HDL particles). Body weight change was inversely associated with glutamate and alanine levels (Factor 10; Table 1)).

**Table 1:** Associations of physical activity and changes in body weight, plasma insulin, glucose and HOMA between baseline and three months after intervention with various factors.

	Regression coefficient	P value
***Factor 1***: ***VLDL subclasses***, ***triglycerides***, ***lipids***		
Physical activity	−0.313 (−0.517 to −0.075)	0.0108
Body weight change	0.294 (0.056 to 0.501)	0.0161
***Factor 7***: ***LDL diameter***, ***medium HDL particles***		
Fasting insulin	0.372 (0.142 to 0.563)	0.0019
Post load glucose	0.300 (0.060 to 0.507)	0.0148
HOMA	0.372 (0.143 to 0.563)	0.0019
***Factor 10***: ***Glutamine***, ***alanine***		
Body weight change	−0.248 (−0.462 to −0.007)	0.0442

The mediation analysis of body weight or fat mass loss on the physical activity-related reductions in the variables of the factor 1 showed a non-significant mediation effect. The proportion of body weight and fat mass to the total effect was mediated by 20.6% and 6.3% on average, indicating that effects of physical activity on biomarkers were 3.9 and 14.8 times greater than those of body weight and fat mass reductions, respectively (Tables 2 and 3).

**Table 2:** Mediation effects of body weight reduction (indirect effect) on the regression between physical activity (direct effect) and the changes in the PCA 1 factor and in its analytes during 3 months’ walking intervention in 67 subjects with high T2D risk.

Dependent variable	Direct effect	Indirect effect	Proportion of totally mediated effect	Sobel-test
	β coefficient	β coefficient		
***PCA Factor 1***	−2.89 (1.09), **p** = **0**.**008**	−0.41 (0.46), p = 0.37	0.12	N.S
***Lipoprotein lipid particles***				
XXL LVDL	−5.7 × 1^−11^ (2.6 × 10^−11^), **p** = **0**.**04**	−1.4 × 10^−12^ (9.4 × 10^−12^), p = 0.24	0.15	N.S
XL VLDL	3.8 × 10^−11^ (1.4 × 10^−12^), **p = 0**.**009**	7.9 × 10^−11^ (5.4 × 10^−11^), p = 0.25	0.14	N.S
L VLDL	−1.8 × 10^−9^ (8.8 × 10^−10^), **p** = **0**.**016**	−6.5 × 10^−10^ (4.1 × 10^−10^), p = 0.23	0.27	N.S
M VLDL	−3.9 × 10^−9^ (2.2 × 10^−9^), **p** = **0**.**046**	−1.7 × 10^−9^ (1.0 × 10^−9^), p = 0.23	0.30	N.S
S VLDL	−5.6 × 10^−9^ (2.4 × 10^−9^), **p = 0**.**021**	−1.2 × 10^−9^ (8.5 × 10^−10^), p = 0.16	0.18	N.S
S HDL	−2.0 × 10^−7^ (8.4 × 10^−8^), **p** = **0**.**017**	−4.63 × 10^−8^ (3.2 × 10^−8^), p = 0.24	0.17	N.S
***Fatty acids and glycoprotein acyl***				
Total fatty acids	−1.49 (0.58), **p** = **0**.**01**	−0.26 (0.26), p = 0.32	0.15	N.S
Monounsaturated	−0.52 (0.21), **p** = **0**.**017**	−0.13 (0.10), p = 0.25	0.20	N.S
Glycoprotein acyl	−0.06 (0.2), **p** = **0**.**021**	−0.02 (0.21), p = 0.28	0.29	N.S

Regression coefficients and p values are given (significant in bold). Lipoprotein lipid abbreviations: XXL = extremely large, XL = very large, L = large, M = medium, S = small. N.S. = non significant.

**Table 3:** Mediation effects of fat mass change (indirect effect) on the regression between physical activity (direct effect) and the changes in the PCA 1 factor and in its analytes during 3 months’ walking intervention in 67 subjects with high T2D risk.

Dependent variable	Direct effect	Indirect effect	Proportion of totally mediated effect	Sobel-test
	β coefficient	β coefficient		
***PCA Factor 1***	−3.14 (0.94), **p** = **0**.**003**	−0.16 (0.32), p = 0.53	0.05	N.S
***Lipoprotein lipid particles***				
XXL LVDL	−6.48 × 1^−11^ (2.6 × 10^−11^), **p** = **0**.**013**	−5.58 × 10^−12^ (9.9 × 10^−12^), p = 0.57	0.08	N.S
XL VLDL	−4.31 × 10^−10^ (1.3 × 10^−10^), **p** = **0**.**001**	−2.62 × 10^−11^ (4.9 × 10^−11^), p = 0.60	0.06	N.S
L VLDL	−2.23 × 10^−9^ (6.8 × 10^−10^), **p** = **0**.**001**	−1.73 × 10^−10^ (3.5 × 10^−10^), p = 0.62	0.07	N.S
M VLDL	−5.20 × 10^−9^ (1.6 × 10^−9^), **p** = **0**.**001**	−4.43 × 10^−9^ (9.2 × 10^−10^), p = 0.63	0.08	N.S
S VLDL	−6.37 × 10^−9^ (2.1 × 10^−9^), **p** = **0**.**002**	−3.79 × 10^−9^ (7.7 × 10^−10^), p = 0.62	0.06	N.S
S HDL	−2.31 × 10^−7^ (7.2 × 10^−8^), **p** = **0**.**001**	−1.34 × 10^−8^ (3.2 × 10^−8^), p = 0.24	0.06	N.S
***Fatty acids and glycoprotein acyl***				
Total fatty acids	−1.67 (0.53), **p** = **0**.**002**	−0.08 (0.18), p = 0.66	0.04	N.S
Monounsaturated	−0.59 (0.20), **p** = **0**.**002**	−0.05 (0.10), p = 0.60	0.08	N.S
Glycoprotein acyl	−0.08 (0.02), **p** = **0**.**001**	−0.003 (0.012), p = 0.82	0.04	N.S

Regression coefficients and p values are given (significant in bold). Lipoprotein lipid abbreviations: XXL = extremely large, XL = very large, L = large, M = medium, S = small, NS = non-significant.

In the subset study the number of daily steps in the highly active group was 2.6 times greater than in the low active group (p < 0.001). After the 3 months’ intervention the changes in baseline and post-load insulin, HOMA, triglycerides and visceral fat area were significantly greater in the highly active than in the low active group. There was no significant difference between plasma ApoD levels in the high and low active groups. The anthropometric and clinical characteristics in this subset are presented in Supplementary Table 1.

**Figure 3:**
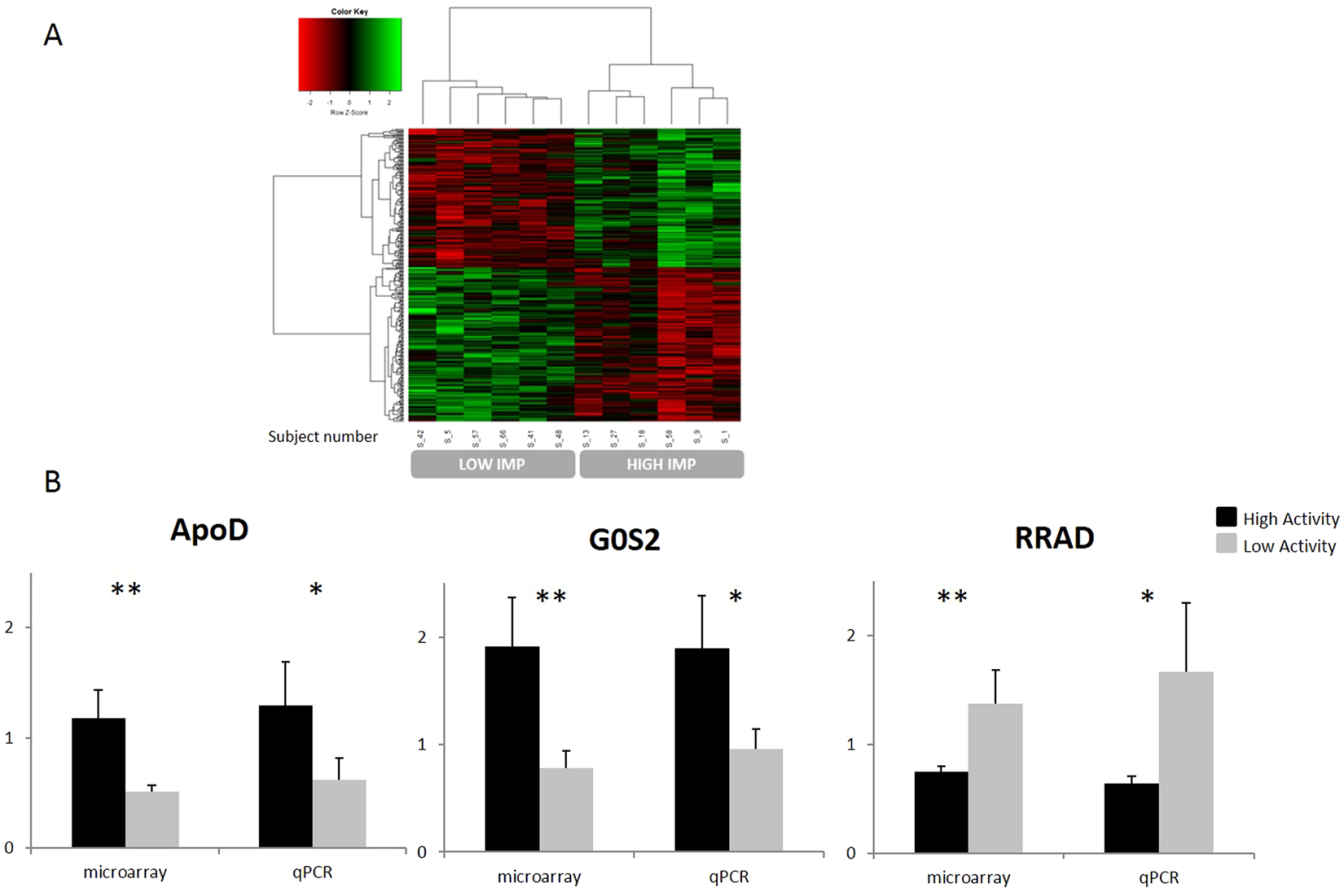
(**A**) Hierarchical clustering analysis of differentially regulated probes between highly active subjects (HIGH IMPACT (IMP)) and low active subjects (LOW IMP). 318 probes that were differentially regulated (p-value < 0.001) were clustered using Ward’s minimum variance method of clustering implemented in hclust library in R. (**B**) Individual qPCR measurements alongside with microarrays values for selected genes of particular interest. Mean Fold change ± SEM are represented for each group. Asterisks mean p value < 0.05.

We then evaluated in a subgroup of subjects their gene expression profile in the quadriceps femoralis muscle. Comparison of the muscular transcriptome’s adaptations using microarray led us to identify 318 probes that clearly segregate the highly active group *versus* the low activity group (Fig. 3A). Expressions of two genes having significant role in lipid metabolism, G0/G1 Switch Gene 2 (GOS2) and Apo D gene, were upregulated 2.3–2.5-fold in the high active subjects compared to low active ones. In the active group we found increased expression of the key gene of insulin action PI3 kinase (1.5-fold) and decreased expressions of the T2D markers RRAD and TGFB3 (0.53–0.54-fold). Furthermore, the expression of the PPPIRC3 gene controlling glycogen synthesis was upregulated (1.7-fold) in the high active subjects.

**Figure 4:**
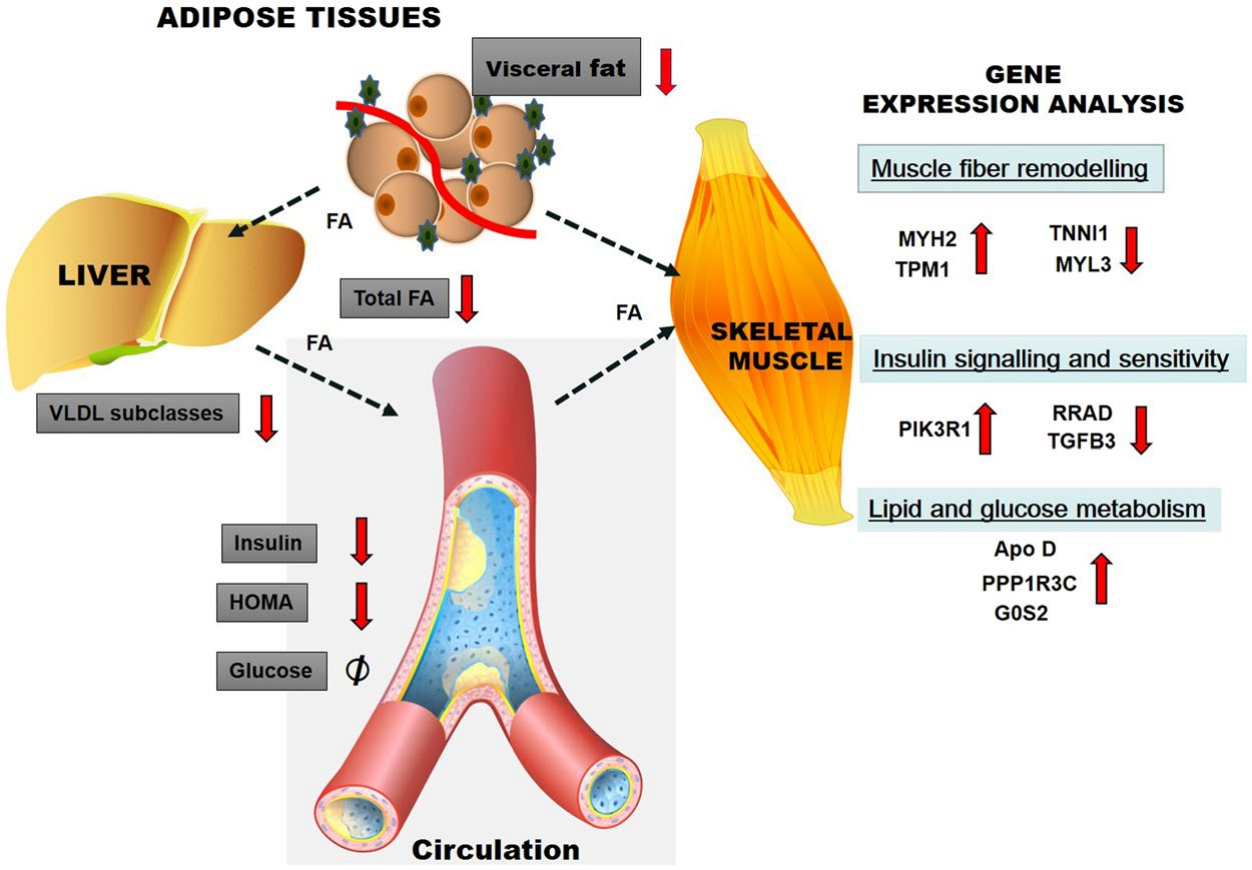
Summary of the results of physical activity intervention on circulating biomarkers and skeletal muscle gene expressions. The intervention included increased physical activity and weight loss leading to reduction of several circulating VLDL and LDL subclasses and fatty acids via decreased hepatic delivery and increased oxidation. Fatty acids were also released from adipose tissues reducing visceral fat mass﻿^8^ and utilized by skeletal muscle for energy production leading up-regulation of fast muscle fiber genes and down-regulation of slow muscle fiber genes. Expression of PPK3 was up-regulated and expressions of TFG3 and RRAD were down-regulated improving insulin sensitivity by decreasing plasma insulin and HOMA. Up-regulation of the APO D expression might be related to decreased circulating lipoprotein lipids and fatty acids and up-regulation of the PPP1R3 and G0S2 to improved formation of muscle cell glycogen from glucose. Downward arrows indicate decrease and upward arrows stimulation; Ø no change.

Validation of microarray results was performed using quantitative PCR approach on ApoD, G0S2 and RRAD genes (Fig. 3B). Both methods produced similar results in gene expression. The gene expression profiles of the most differentially regulated probes are shown in Supplementary Table 3. Tropomyosin I and Myosin H2 known to be present in type II fast fibers were increased by 2.7–2.8-fold in the group with high activity, whereas Troponin 1 and Myosin L3 and tropomyosin 3, markers of slow type I fibers, were down-regulated by 0.61–0.66-fold. A summary of the NMR and gene expression results is presented as a model in Fig. 4.

## Discussion

We have previously demonstrated within a large scale of ambulatory activities significant health effects of objectively measured amounts and intensities of whole - day physical activities. In healthy women approximately 1000 steps daily at walking speed of 5 km/h was associated with maximal oxygen uptake and reduced total and LDL cholesterol^15^. In our study consisting of sedentary and obese subjects with high T2D risk, daily 5500–6500 steps at walking speed of 2–3 km/h reduced basal and post-challenge insulin, HOMA, triglycerides, total and LDL cholesterol and visceral fat in three months^8^. Here, we further investigated circulating NMR biomarkers in these subjects. After the intervention several VLDL particle concentrations (extremely large, very large, large medium and small), triglycerides (VLDL, extremely large VLDL), fatty acids (total, monounsaturated, ω-7 and ω-9), glycoprotein acetyl (GlycA), small HDL particles and phospho-glycerides were significantly decreased in subjects walking more than 2895 steps daily at speed 2–3 km/h.

This finding was unexpected, since in the same subjects the decreases in total and LDL cholesterol took place when daily steps exceeded 6500^8^. Reductions in circulating VLDL subclass particles and lipids in response to exercise are in part associated to lipid oxidation that occurs at the highest level of 30–40% of the maximal oxygen uptake in laboratory conditions^16^. In our sedentary subjects the maximal lipid oxidation appears to emerge at the level of 2895 daily steps with no further changes at higher daily step levels. This number of daily steps at the acceleration level of 1.3–1.7 g (e.g. slow walking 2–3 km/h) corresponds to the energy expenditure of 10–14 MET h/week^8^. This amount could be very feasible in the planning of physical activity programs addressing cardio-metabolic health and weight reduction.

Of the life style factors body weight gains and losses have claimed to be strongly associated with VLDL, LDL and large HDL subclasses, triglycerides, amino acids and metabolic markers^17–19^. However, in these studies, only little or no attention has been paid to physical activity as objective recordings were not performed. In the present study quantitative measures of physical activity and body weight were available and their associations with health markers could be analyzed for the first time. Our subjects lost 1.1 kg body weight on average during 3 months’ intervention^8^ and a positive correlation between weight changes and factor 1 variables was observed. The observed body weight reduction was mainly related to body fat while skeletal muscle mass did not change. Therefore, we were able to investigate the mediating effects of this body weight loss on the lipoprotein and lipid profiles. The relative effect of physical activity was 4–15 times greater than that of body weight or fat mass reduction in this intervention study. Thus, for recommendations of healthy life style habits increased physical activity appears to play a greater role than body weight reduction.

Negative associations between physical activity and VLDL subclasses, triglycerides and lipids and positive associations with body weight change have been widely reported previously^4–7, 17–19^ resulting in beneficial health effects^2, 3^. The negative association between body weight change and changes in plasma glutamine and alanine was unexpected, since in one cross-sectional population study a positive association was found^19^. The physical activity intervention we used here might have led to better association with the metabolites than in the cross-sectional study.

We observed that LDL particle sizes showed positive associations with basal insulin, 2 h glucose and HOMA in agreement of previously studies in which low LDL size associated with decreased glucose disposal rate^10^ and increased risk for cardiovascular diseases^11^.

In the subset study significant changes in the transcription of several skeletal muscle genes related to muscle fiber types and glucose and lipid metabolism were observed. Interestingly, amongst the most differentially regulated genes were several type I or II fibers markers. Tropomyosin I and myosin H2 (markers of type II fast fibers)^20^ increased in the group with high activity, whereas troponin 1, myosin L3 and tropomyosin 3 (markers of slow type I fibers)^21, 22^ were down-regulated in response to high activity, suggesting remodeling of the fiber composition. This fiber remodeling, improving regular muscle contractions, appears to require physical activity of at least 8000 daily steps. The 2.7–2.8-fold increase in the expression of the type 1 fibers may also lead to increased mitochondrial fatty acid oxidation explaining the reductions in the lipoprotein lipids and fatty acids, which we observed after the intervention^23^.

The 2.3-foldly increased expression of Apo D gene in response to high physical activity is an interesting finding. Epidemiological studies have shown that missense mutations of the Apo D gene are associated with elevated plasma triglycerides^24, 25^ and a *Taq1* polymorphism with the development of obesity, insulin resistance and T2D^26, 27^. In mice treated with an APO D vector an improved triglyceride profile and increased lipoprotein lipase activity have been observed and to be associated with increased VLDL triglyceride hydrolysis and clearance^28^. In contrast, Apo D knockout mice develop hypertriglyceremia and hyperinsulinemia, supporting the genetic mutations studies^29^. Thus, Apo D gene variants and lack of Apo D proteins seem to be associated with abnormal lipid metabolism and increased risk for T2D. The protecting role of Apo D has been documented especially in nerve injuries and counted for its capability to protect cells from oxidative stress. However, in an earlier study an increased Apo D gene expression was found in non-active muscles^30^ contrasting with our observation. Further studies are thus needed to clarify the potential role of Apo D in muscle in beneficial actions of physical activity.

G0/G1 switch gene 2 expression, another gene involved in lipid metabolism, was 2.5-fold upregulated in active subjects. In the adipose tissue this gene controls lipolysis via the triglyceride lipase and its invalidation in mice generated increased lipolysis, gluconeogenesis and improved insulin sensitivity^31^. In human subjects one hour exercise did not change the expression of the GOS2 in the adipose tissue^32^. Our results indicate that increased physical activity for 3 months stimulates the expression of GOS2 in skeletal muscle together with improved circulating lipid status and insulin sensitivity.

The increased expression of the key gene of insulin action PI3 kinase with decreased expressions of T2D markers RRAD and TGFB3 in our highly active group are in agreement with decreased plasma insulin and improved insulin sensitivity and support the beneficial effects of high physical activity on insulin secretion. In addition, the product of the PPP 13RC gene regulates glycogen synthase enzyme^33^ and thus its up-regulation in the highly active subjects results in improved glucose metabolism in skeletal muscles. Overall, our findings indicate that increased physical activity improves at molecular level many aspects of insulin and glucose metabolism.

We observed that changes between baseline and 3 months in GlycA concentrations were significantly lower in the first quartile (daily steps <2895) than in other quartiles and followed those we observed in large VLDL particles. This is in agreement with a previous finding in which GlycA levels were lower in physically highly active than in inactive subjects^7^. We extend here this finding demonstrating that physical activity-induced decrease in ClycA occurs already at a very low level. Produced by liver it circulates in micromolar concentrations acting as an acute-phase reactant protein. Urinary excretion of GlycA has been also observed to be elevated in patients with T2D and to independently predict T2D, cardiovascular and all-cause mortality^34^. Molecular profiling of biobank blood samples by NMR spectroscopy have revealed that high GlycA levels with three other markers are predictors for cardiovascular, cancer and all-cause deaths^35^. Recently it was demonstrated that increased levels of GlycA are associated with chronic inflammation, neutrophil activity and risk of severe infections in future^36^. These findings support previous large epidemiological studies demonstrating that minimum amounts of physical activity (15–30 min daily) are associated with a reduce mortality and extended life expectancy^37, 38^.

Our investigation has several limitations: The sample size was small, but taking blood samples and biopsies before and after the intervention in the same subjects reduced variability. Use of PCA collapsed the experimental data and at lower factor loads clinically relevant findings may have remained unnoticed. In population studies sample sizes easily extend from hundreds to thousands are impossible to be realized in intervention trials.

The strength of our study was the registration of total ambulatory energy expenditure during the whole intervention, allowing the determination of physical activity thresholds for beneficial health outcomes and proportional effects of life style factors.

The present study focused on the effects of total ambulatory energy expenditure on lipoprotein lipid particles, triglyceride and fatty acid measures in subjects with high T2D risk which demonstrated interesting and previously unknown observations. First, the mediating effects of the body weight loss on NMR biomarkers were not significant, but those of physical activity were. It is evident that quantitative recordings of the magnitudes of life style factors are required in assessing of their health benefits. Secondly, the unexpectedly low energy expenditure exceeding 2895 steps/day or 10–14 MET h/week attenuated the concentrations of wide scale of important biomarkers suggesting their high sensitivity to energy expenditure. Therefore, the threshold number of daily steps 2895 we documented here can be used as an amount of physical activity which might prove useful for improving lipid metabolism and preventing risks for cardio-metabolic diseases and for weight reduction programs in sedentary and overweight subjects with impaired glucose metabolism. Furthermore, increased physical activity was found to lead to changes in the gene expressions of muscle fiber, Apo D and G0/G1 S 2 proteins controlling fatty acid oxidation, lipid metabolism, insulin signaling and glucose uptake and utilization, demonstrating that low amounts of physical activity have significant effects on our transcriptome.

## Methods

The present study investigated the effect of physical activity on circulating NMR biomarkers in 67 subjects with impaired glucose metabolism (IGM) and in a subset of 20 subjects who were willing to undergo additional muscle biopsies its influence on muscle gene expressions (Fig. 1). The study design of the main study with the inclusion of 67 overweight and sedentary males and females originally recruited from screened 113 subjects with a high risk of T2D have been described previously^8^. All subjects completed the FINDRISC questionnaire for T2D (http://www.idf.org/webdata/docs/FINDRISC_English.pdf20). Subjects with scores >15 were further evaluated by an oral glucose tolerance test. The WHO criteria for impaired fasting glucose (≥5.6 and <7.0 and 2 h glucose <7.8 mmol l^−1^) or impaired glucose tolerance (fasting glucose <7.8 and 2 h glucose ≥7.8 and <11.1 mmol l^−1^) were used. 35 subjects of those were excluded for normoglycemia or overt T2D and 11 were drop-outs or missing data/samples. Major medical comorbidities were hypertension and hypercholesterolemia: 36 subjects were on antihypertensive medication and 24 on lipid-lowering drugs. The purpose of the original study was to determine physical activity thresholds improving glucose and lipid metabolism. 67 subjects were distributed by computer based randomization by age, gender and medication to intervention and control groups. The significance of regular exercise and weight reduction as the best standard care to prevent T2D was addressed in three common meetings by authors (SKK, KHH, JL) and by a leaflet distributed before the intervention. During the 3 months’ intervention subjects participated in structured and supervised walking exercise 3 times weekly consisting of a 5–10 min warming-up followed by a 40–50 min walking at the speed of 2–3 km/h and muscle relaxation of 5 min in an indoor hall while the control group continued their usual physical activities. The dietary intake of the participating subjects was monitored by 3-day dietary questionnaire under the supervision of a professional dietician before and after the intervention. All subjects carried accelerometers (Newtest, Finland) during the whole wakeful time for three months (mean wear time was 78 days). The Newtest device is a uniaxial accelerometer with sampling rate of 400 per second and registers accelerations from 1.3 to 10.9 (g = 9.81 m/s2, 1 g presents standing). The reproducibility error of the peak accelerations (root-mean square of coefficient of variation) was 4.0% and correlations of acceleration values with simultaneously used optical motion analysis or with Kistler force plate were high (R = 0.989 and 0.937, respectively^39^). The variation in the weighted average of accelerations in walking/running speeds of 3, 6 and 9 km/h accounted for 92% of the variance in energy expenditure as measured in oxygen consumption tests on treadmill^8^. The physical activity of each subject was determined as the average number of daily steps during the 3 months’ intervention. All subjects met weekly the research assistants for downloading accelerometer data. Weekly MET/h were calculated using codes 17150 and 17151^40^ representing 2 METs and walking time 2895 steps/day at 1.3–1.7 g of 1–1.5 hrs.

Visceral adipose tissue was measured by bioelectrical impedance analysis (InBody 720, Mega Electronics, Kuopio, Finland).

In a subset study 20 subjects from the main study of 67 subjects volunteered for additional muscle biopsies before and after the intervention. Valid samples were obtained from 12 subjects (6 drop-outs and two technical failures) and divided to high and low active subjects based on the numbers of daily steps.

The protocol was approved by the local institutional ethics committee (Northern Ostrobothnia Hospital District 113/2009) and in compliance with national legislation and the Declaration of Helsinki. All subjects gave their informed written consent.

### Circulating metabolite measurements

Fasting EDTA plasma was collected between 8 and 10 am before and after the intervention, stored −72 °C and analyzed using a high throughput NMR metabolomics platform providing information on lipoprotein subclasses, lipids, cholesterol, amino acids ketones, glycolytic precursors and proteins^41^.

### Muscle biopsies and gene expression analysis

Muscle biopsies of a subgroup of subjects willing to participate were divided to high activity and low activity groups (ranges 6946–11735 and 2309–4637 daily steps, respectively). They were taken before and after the intervention by the Bergström needle under local anesthesia in the left anterior part of the quadriceps femoralis muscle. Valid muscle samples from 12 subjects before and after the intervention were available. Metabolic and anthropological parameters before and after a 3-month physical activity intervention in the subjects are presented in Supplementary Table 1.

RNA profiling in skeletal muscle biopsies was performed using Agilent Human whole genome 4 * 44 K V2 after isolating of total RNA. RNA was submitted to the Low Input Quick Amp labeling two color kit (Agilent). Dye incorporation and antisense RNA (aRNA) yield were checked and Cy3 & Cy5 dye-labelled aRNA was fragmented and hybridized. Slides were scanned and the images were analyzed with Feature Extraction Software 10.5 (Agilent). Data were normalized using R and limma library. Data have been deposited in NCBI’s Gene Expression Omnibus and are accessible through GEO Series accession number GSE62524. First-strand cDNAs were synthesized from 1 µg of total RNAs using PrimeScript RT kit (Ozyme, Saint-Quentin-en-Yveline, France) with a mixture of random hexamers and oligo(dT) primers and treated with 60 units of RnaseH (Ozyme) Real-time PCR assays were performed with Rotor-Gene 6000 (Qiagen, Courtaboeuf, France) for ApoD, G0S2 and PRAD. List of the primers and real-time PCR assay conditions are available upon request. The results were normalized by using TBP (TATA box binding protein) mRNA concentration, measured as reference gene in each sample. A-hierarchical clustering analysis of differentially regulated probes between highly active subjects (HIGH IMP) and low active subjects (LOW IMP) were clustered using Ward’s minimum variance method of clustering implemented in hclust library in R.

Plasma ApoD concentrations of the biopsy subjects were measured by a commercial ELISA (Human ApoD ELISA PRO kit; Mabtech AB, Nacka Strand, Sweden).

### Statistical analyses

Associations between physical activity, body weight, conventionally measured analytes^8^ and changes in plasma concentration of lipoprotein lipids, lipids and metabolic intermediates during 3 months’ intervention were studied by principal component analysis (PCA) to reduce the complexity of the NMR platform variables. The analysis produced 11 factors (Supplementary Table 2). Spearman correlations between physical activity (daily steps) and the factors were calculated. Variables in each factor were further divided into quartiles (Q1–4) according to physical activity levels (1^st^ below 2895, 2^nd^ below 4150, 3^rd^ below 7000 and 4^th^ over 7100 daily steps). The differences in variable changes between the 1^st^ quartile and other quartiles were analyzed by using bootstrapping procedure creating 5000 replicas for means and standard deviations with 95% confidential limits. Data were adjusted to baseline, age and gender. For the 16 variables of the factor 1 the significance level was set at 0.0036. The mediation effects of body weight or fat mass change on the physical activity-related changes in the biomarkers were studied by Sobel-Goodman method using bootstrapping with 5000 replications^42^. We analyzed associations between dependent outcome variables, exposure and statin treatment in PA quartiles and no significant associations were found (data not shown).

In the subset study muscle gene expression data was normalized using R and limma library on 43.376 probes with moderate t-statistics and given as a ratio between expressions at 3 and 0 months. Probes presenting a ratio with a p value <0.001 were considered as differentially regulated between high and low activity groups. Conventional and anthropological data were analyzed by SPSS statistical package (SPSS Inc. Chicago, IL, USA) using repeated measures ANOVA, unpaired t-test and Mann-Whitney nonparametric test.

## Electronic supplementary material

Supplementary information

Supplementary Table 1

Supplementary Table 2

Supplementary Table 3
