# Supplementary material for: Low level activity thresholds for changes in NMR biomarkers and genes in high risk subjects for Type 2 Diabetes
Source: Sci Rep. 2017 Sep 18;7:11267. doi: 10.1038/s41598-017-09753-6 (PMC5603534; doi:10.1038/s41598-017-09753-6)
Supplement: Supplementary file 1 — Supplementary information [file 41598_2017_9753_MOESM1_ESM.doc]

**Supplementary information**

to

**Low level activity thresholds for changes in NMR biomarkers and genes** **in high risk subjects** **for Type 2 Diabetes**

Authors:

Karl-Heinz Herzig, MD, PhD, Juhani Leppäluoto, MD, PhD, Jari Jokelainen, PhD, Emmanuelle Meugnier, PhD, Sandra Pesenti, PhD, Harri Selänne, MD, PhD, Kari A. Mäkelä, Riikka Ahola, PhD, Timo Jämsä, PhD, Hubert Vidal, MD, PhD, Sirkka Keinänen-Kiukaanniemi, MD, PhD

| Supplementary Table 1 | **Metabolic and anthropological parameters before and after a 3-month physical activity intervention**  **in high and low activity subjects with muscle biopsies (N=12)** | | | | | | | | | | | | | | | | | |
| --- | --- | --- | --- | --- | --- | --- | --- | --- | --- | --- | --- | --- | --- | --- | --- | --- | --- | --- |
|  |  | |  | |  | |  |  |  |  |  | |  |  |  |  |  |  |
| **Plasma concentrations** | High activity | | | | (n = 7) | | | | Low activity | | (n= 5) | | |  |  |  |  |  |
| **and** | *Base-line* | |  | | *At 3 months* | | |  | *Base-line* |  | *At 3 months* | | |  |  |  |  |  |
| **body constituents** | mean | | SD | | mean | | SD | p | mean | SD | mean | | SD | p | Difference | |  | P |
| Fasting glucose (mmol l^-1^) | **7.22** | | 1.01 | | **7.07** | | 0.69 | *0.44* | **6.47** | 0.69 | **6.48** | | 0.79 | *0.91* | -0.17 | (-0.26 to 0.59) | | 0.44 |
| 2 h glucose mmol l-1) | **9.70** | | 3.62 | | **8.60** | | 3.60 | *0.21* | **8.35** | 2.12 | **7.18** | | 2.35 | *0.11* | 0.09 | (-1.88 to 1.74) | | 0.94 |
| Fasting insulin mU l^-1^ | **20.50** | | 14.22 | | **14.33** | | 12.27 | *0.08* | **12.67** | 7.66 | **14.33** | | 6.62 | *0.35* | 3.20 | (1.56 to 14.1) | | **0.02** |
| 2 h insulin mU l-1) | **111.7** | | 90.95 | | **63.2** | | 38.57 | *0.10* | **117.2** | 82.70 | **107.7** | | 73.07 | *0.60* | -28.6 | (-17.0 to 95.0) | | 0.17 |
| HOMA IR | **6.94** | | 6.01 | | **4.72** | | 4.68 | *0.07* | **3.72** | 2.49 | **4.19** | | 2.11 | *0.44* | -1.05 | (-0.61 to -4.74) | | **0.01** |
| Cholesterol mmol l-1) | **5.15** | | 0.47 | | **4.90** | | 0.78 | *0.36* | **5.85** | 1.46 | **5.55** | | 1.58 | *0.21* | -0.32 | (-0.67 to 0.57) | | 0.88 |
| HDL cholesterol mmol l-1) | **1.44** | | 0.34 | | **1.50** | | 0.54 | *0.58* | **1.54** | 0.41 | **1.52** | | 0.43 | *0.79* | 0.11 | (-0.29 to 0.14) | | 0.5 |
| LDL cholesterol mmol l-1) | **2.85** | | 0.57 | | **2.90** | | 0.26 | *0.84* | **3.20** | 1.13 | **3.39** | | 1.41 | *0.41* | -0.31 | (-0.47 to 0.75) | | 0.44 |
| Triglycerides (mmol l^-1^) | **1.43** | | 0.53 | | **1.15** | | 0.33 | *0.13* | **2.17** | 1.03 | **2.07** | | 1.25 | *0.53* | -0,21 | (-0.24 to 0.60) | | 0.83 |
| ApoD (ug/l) | **123** | | 19,0 | | **123** | | 28.0 | *0.99* | **115** | 14.8 | **105** | | 8.2 | *0.14* | -10.0 | (-22.2 to -52.8) | | 0.57 |
| Weight (kg) | **85.48** | | 23.11 | | **83.42** | | 23.68 | *0.07* | **88.30** | 12.06 | **88.06** | | 12.57 | *0.37* | -0.96 | (-0.33 to -4.09) | | **0.02** |
| BMI (kg m^-2^) | **30.22** | | 6.03 | | **29.47** | | 6.30 | *0.09* | **29.64** | 4.57 | **29.58** | | 4.71 | *0.43* | -0.34 | (-0.03 to -1.39) | | **0.04** |
| Waist circumference (cm) | **91.83** | | 16.88 | | **91.67** | | 16.79 | *0.94* | **97.20** | 11.30 | **92.50** | | 5.43 | *0.19* | -4.52 | (-3.00 to -7.85) | | 0.29 |
| Fat% | **38.02** | | 8.43 | | **33.13** | | 10.29 | *0.2* | **32.72** | 11.01 | **33.34** | | 11.93 | *0.48* | -0.40 | (-0.99 to -14.9) | | 0.09 |
| Visceral fat area (cm^2^) | **161.6** | | 59.5 | | **141.1** | | 39.4 | *0.09* | **161.9** | 22.65 | **163.5** | | 21.50 | *0.62* | -9.90 | (-3.83 to -42.6) | | **0.02** |
| Daily steps were averaged from the 3 months period. High active subject walked 8539±1942 (mean ±SD) and low ones 3463±981 steps per day. | | | | | | | | | | | | | | | | | | |
| *p for within group changes* | |  | |  | |  | | | | | |  | | | | | | |
| *P for difference in change between groups* | | | | | |  | | | | | |  | | | | | | |
| 12 subjects (3 females in both groups) were studied before and after the intervention | | | | | | | | | | | |  | | | | | | |

**Suppl Tab. 2**: Principal component analysis of the metabolome measures. Rotated factor pattern was used, values less than 0.3 were excluded.

**Factor1 Factor2 Factor3 Factor4 Factor5 Factor6 Factor7 Factor8 Factor9 Factor10 Factor11**

**Large VLDL particles 0.96331 . . . . . . . . . .**

**Triglycerides in VLDL 0.95369 . . . . . . . . . .**

**Medium VLDL particles 0.94752 . . . . . . . . . .**

**Serum Triglycerides 0.93018 . . . . . . . . . .**

**Very large VLDL particles 0.92588 . . . . . . . . . .**

**Extremely large VLDL particles 0.88236 . . . . . . . . . .**

**Median Diameter of VLDL 0.85798 . . . . . . . . . .**

**Trigly. in Extremely large VLDL 0.84238 . . 0.33010 . . . . . . .**

**Small VLDL particles 0.81538 0.43904 . . . . . . . . .**

**ω7/ω9 fatty acids 0.81286 0.44119 . . . . . . . . .**

**Monounsaturated fatty acids 0.81178 0.40600 . . . . . . . . .**

**Glycoprotein acyls 0.78750 . . . . . . . . . .**

**Total fatty acids 0.77141 0.53955 . . . . . . . . .**

**Small HDL particles 0.66464 . . . . . . . -0.30555 . .**

**Total Cholesterol in HDL3 0.54417 . 0.32897 . . . . . . . .**

**Total Phosphoglycerides 0.52146 0.42889 . . . . . . . . .**

**Large LDL particles . 0.98872 . . . . . . . . .**

**Total cholesterol in large LDL . 0.98860 . . . . . . . . .**

**Total cholesterol in LDL particles. 0.97127 . . . . . . . . .**

**Total Serum Cholesterol . 0.96905 . . . . . . . . .**

**Total cholesterol in IDL particles. 0.95367 . . . . . . . . .**

**Medium LDL particles . 0.94618 . . . . . . . . .**

**IDL particles . 0.93233 . . . . 0.31979 . . . .**

**Small LDL particles 0.30652 0.87956 . . . . . . . . .**

**Triglycerides in IDL particles 0.35601 0.75729 . . . . 0.37682 . . . .**

**Very small VLDL particles . 0.74465 . . . . 0.55179 . . . .**

**ω6 fatty acids 0.46788 0.72303 . . . . . . . . .**

**Apolipoprotein B / ApolipoA1 0.33317 0.66737 -0.56917 . . . . . . . .**

**Phosphatidylcholine& cholines 0.53277 0.60159 . . . . . . . . .**

**Total cholesterol in HDL part. . . 0.96071 . . . . . . . .**

**Total cholesterol in HDL2 part. . . 0.93110 . . . . . . . .**

**Large HDL particles . . 0.93000 . . . . . . . .**

**Total cholesterol in Large HDL . . 0.89037 . . . . . . . .**

**Mean diameter of HDL particles . . 0.86595 . . . . . . . .**

**Extra large HDL particles . . 0.72213 . . . -0.39781 . . . .**

**Valine . . . 0.87153 . . . . . . .**

**Leucin 0.37773 . . 0.87142 . . . . . . .**

**Tyrosine . . . 0.76243 . . . . . 0.31457 .**

**Isoleucine 0.54006 . . 0.70852 . . . . . . .**

**Phenylalanine . . . 0.70672 . . . . . . .**

**3-hydroxybutrate . . . . 0.86531 . . . . . .**

**Acetoactate . . . 0.36634 0.82459 . . . . . .**

**Glucose . . . . 0.69983 . . . . . -0.31201**

**Acetate . . . . 0.57484 . . . . . 0.33755**

**ω3 fatty acids . . . . . 0.85389 . . . . .**

**Double bonds in fatty acids -0.62918 . . . . 0.65313 . . . . .**

**Methylene groups/double bonds 0.61766 . . . . -0.65560 . . . . .**

**Mean diameter for LDL particles -0.40239 . . . . . 0.85036 . . . .**

**Medium HDL particles 0.45682 . 0.45785 . . . 0.47944 . . . .**

**Lactate . . . . . . . 0.80609 . . .**

**Glycerol . . . . . . . 0.76228 . . .**

**Pyruvate 0.41859 . . . . . . 0.61290 . . .**

**Citrate . . . . 0.31455 . . 0.47894 0.30039 . 0.32173**

**Histidine . . . . . . . . 0.76782 . .**

**Glycine . . . . . . . . 0.74479 . .**

**Glutamine . . . . . . . . 0.38789 0.68226 .**

**Alanine 0.31827 . . . -0.30012 . . 0.47111 . 0.57999 .**

**Urea . . . . . . . . . . 0.76159**

**Creatinine . . . . 0.34351 0.30339 . . . . 0.45061**

**Values less than [0.3] are not printed.**

SupplementaryTable 3: List of the most increased (A) and most downregulated genes (B) of subjects with high physical activity

compared to those with low activity

| **ProbeName** | **raw**  **p- value** | **Fold Change in High Activity group** | **Fold Change in Low Activity Group** | **Ratio HIGH/LOW activity** | **Gene**  **Symbol** | | **Gene name** | |
| --- | --- | --- | --- | --- | --- | --- | --- | --- |
|  | 1. **Most upreguated genes** | | | | | | | |
| A_23_P38271 | 0.00385 | 1.562 | 0.567 | 2.754 | **MYH2** | | myosin, heavy chain 2, skeletal muscle, adult | |
| A_23_P363344 | 0.00624 | 1.588 | 0.586 | 2.708 | **TPM1** | | tropomyosin 1 (alpha) | |
| A_23_P74609 | 0.00383 | 1.914 | 0.779 | 2.456 | **G0S2** | | G0/G1switch 2 | |
| A_23_P136777 | 0.00083 | 1.179 | 0.508 | 2.323 | **APOD** | | apolipoprotein D | |
| A_23_P35414 | 0.0048 | 1.141 | 0.661 | 1.726 | **PPP1R3C** | | protein phosphatase 1, regulatory subunit 3C | |
| A_24_P76210 | 0.00313 | 1.149 | 0.682 | 1.685 | **A_24_P76210** | | Unknown | |
| A_23_P344515 | 0.00315 | 1.35 | 0.804 | 1.679 | **C16orf3** | | chromosome 16 open reading frame 3 | |
| A_24_P44462 | 0.0033 | 1.194 | 0.715 | 1.67 | **TPM1** | | tropomyosin 1 (alpha) | |
| A_32_P115518 | 0.00079 | 1.315 | 0.792 | 1.661 | **AI207522** | | HA2878 Human fetal liver cDNA library  Homo sapiens cDNA | |
| A_24_P926849 | 0.00112 | 1.346 | 0.834 | 1.615 | **AJ230821** | | clone PS14C5 | |
| A_32_P78101 | 0.00105 | 1.095 | 0.689 | 1.589 | **IGSF21** | | immunoglobin superfamily, member 21 | |
| A_23_P36985 | 0.00413 | 1.193 | 0.762 | 1.567 | **PCDH8** | | protocadherin 8 | |
| A_24_P319675 | 0.00419 | 1.257 | 0.811 | 1.549 | **RAB10** | | RAB10, member RAS oncogene family | |
| A_23_P144980 | 0.00225 | 1.207 | 0.783 | 1.542 | **PIK3R1** | | phosphoinositide-3-kinase,  regulatory subunit 1 (alpha) | |
| A_24_P522678 | 0.00186 | 1.076 | 0.702 | 1.533 | **AF117899** | | Homo sapiens LDLR-FUT fusion protein (LDLR-FUT) | |
| A_23_P201287 | 0.00737 | 1.248 | 0.814 | 1.533 | **KIF1B** | | kinesin family member 1B | |
| A_23_P121253 | 0.00775 | 1.29 | 0.842 | 1.533 | **TNFSF10** | | tumor necrosis factor (ligand) superfamily,  member 10 | |
| A_24_P579439 | 0.0079 | 1.059 | 0.696 | 1.522 | **AF086790** | | aconitase precursor | |
| A_23_P24004 | 0.00744 | 1.419 | 0.932 | 1.521 | **IFIT2** | | interferon-induced protein with tetratricopeptide  repeats 2 | |
| A_32_P160186 | 0.00369 | 1.165 | 0.768 | 1.517 | **EIF5** | | Eukaryotic translation initiation factor 5 | |
| **B) Most downregulate genes** | | | | | | | |  |
| A_23_P115261 | 0.00221 | 0.977 | 1.305 | 0.749 | **AGT** | angiotensinogen (serpin peptidase inhibitor,  clade A, member 8) | |  |
| A_32_P96807 | 0.00294 | 0.871 | 1.169 | 0.745 | **RC3H1** | ring finger and CCCH-type domains 1 | |  |
| A_23_P154585 | 0.00538 | 1.038 | 1.393 | 0.745 | **SNX21** | sorting nexin family member 21 | |  |
| A_23_P433753 | 0.00589 | 0.933 | 1.264 | 0.738 | **PRKAR1A** | protein kinase, cAMP-dependent, regulatory,  type I, alpha (tissue specific extinguisher 1) | |  |
| A_32_P62863 | 0.00099 | 0.86 | 1.183 | 0.727 | **SCHIP1** | schwannomin interacting protein 1 | |  |
| A_23_P132468 | 0.00574 | 0.91 | 1.251 | 0.727 | **SLC4A7** | solute carrier family 4, sodium bicarbonate cotransporter,  member 7 | |  |
| A_23_P132644 | 0.00813 | 1.016 | 1.397 | 0.727 | **NCEH1** | neutral cholesterol ester hydrolase 1 | |  |
| A_23_P131202 | 0.00242 | 0.831 | 1.175 | 0.707 | **HES6** | hairy and enhancer of split 6 (Drosophila) | |  |
| A_23_P47704 | 0.00786 | 0.842 | 1.262 | 0.667 | **UCP2** | uncoupling protein 2 (mitochondrial,  proton carrier) | |  |
| A_24_P67364 | 0.0062 | 0.87 | 1.318 | 0.66 | **TPM3** | tropomyosin 3 | |  |
| A_24_P131646 | 0.00173 | 0.885 | 1.347 | 0.657 | **MYL3** | myosin, light chain 3, alkali; ventricular,  skeletal, slow | |  |
| A_23_P55846 | 0.00654 | 0.885 | 1.348 | 0.656 | **LOC147804** | tropomyosin 3 pseudogene | |  |
| A_32_P80255 | 0.00327 | 0.851 | 1.308 | 0.65 | **DDX6** | DEAD (Asp-Glu-Ala-Asp) box helicase 6 | |  |
| A_24_P249253 | 0.00941 | 0.899 | 1.422 | 0.632 | **LMOD2** | leiomodin 2 (cardiac) | |  |
| A_23_P51565 | 0.00454 | 0.865 | 1.402 | 0.617 | **TNNI1** | troponin I type 1 (skeletal, slow) | |  |
| A_23_P155638 | 0.0015 | 0.862 | 1.407 | 0.613 | **MYL3** | myosin, light chain 3, alkali; ventricular,  skeletal, slow | |  |
| A_23_P88849 | 0.00913 | 0.743 | 1.368 | 0.543 | **RRAD** | Ras-related associated with diabetes | |  |
| A_23_P88404 | 0.003 | 0.997 | 1.888 | 0.528 | **TGFB3** | transforming growth factor, beta 3 | |  |
