# Supplementary material for: Low level activity thresholds for changes in NMR biomarkers and genes in high risk subjects for Type 2 Diabetes
Source: Sci Rep. 2017 Sep 18;7:11267. doi: 10.1038/s41598-017-09753-6 (PMC5603534; doi:10.1038/s41598-017-09753-6)
Supplement: Supplementary file 2 — Supplementary Table 1 [file 41598_2017_9753_MOESM2_ESM.doc]

| Supplementary Table 1 | **Metabolic and anthropological parameters before and after a 3-month physical activity intervention**  **in high and low activity subjects with muscle biopsies (N=12)** | | | | | | | | | | | | | | | | | |
| --- | --- | --- | --- | --- | --- | --- | --- | --- | --- | --- | --- | --- | --- | --- | --- | --- | --- | --- |
|  |  | |  | |  | |  |  |  |  |  | |  |  |  |  |  |  |
| **Plasma concentrations** | High activity | | | | (n = 7) | | | | Low activity | | (n= 5) | | |  |  |  |  |  |
| **and** | *Base-line* | |  | | *At 3 months* | | |  | *Base-line* |  | *At 3 months* | | |  |  |  |  |  |
| **body constituents** | mean | | SD | | mean | | SD | p | mean | SD | mean | | SD | p | Difference | |  | P |
| Fasting glucose (mmol l^-1^) | **7.22** | | 1.01 | | **7.07** | | 0.69 | *0.44* | **6.47** | 0.69 | **6.48** | | 0.79 | *0.91* | -0.17 | (-0.26 to 0.59) | | 0.44 |
| 2 h glucose mmol l-1) | **9.70** | | 3.62 | | **8.60** | | 3.60 | *0.21* | **8.35** | 2.12 | **7.18** | | 2.35 | *0.11* | 0.09 | (-1.88 to 1.74) | | 0.94 |
| Fasting insulin mU l^-1^ | **20.50** | | 14.22 | | **14.33** | | 12.27 | *0.08* | **12.67** | 7.66 | **14.33** | | 6.62 | *0.35* | 3.20 | (1.56 to 14.1) | | **0.02** |
| 2 h insulin mU l-1) | **111.7** | | 90.95 | | **63.2** | | 38.57 | *0.10* | **117.2** | 82.70 | **107.7** | | 73.07 | *0.60* | -28.6 | (-17.0 to 95.0) | | 0.17 |
| HOMA IR | **6.94** | | 6.01 | | **4.72** | | 4.68 | *0.07* | **3.72** | 2.49 | **4.19** | | 2.11 | *0.44* | -1.05 | (-0.61 to -4.74) | | **0.01** |
| Cholesterol mmol l-1) | **5.15** | | 0.47 | | **4.90** | | 0.78 | *0.36* | **5.85** | 1.46 | **5.55** | | 1.58 | *0.21* | -0.32 | (-0.67 to 0.57) | | 0.88 |
| HDL cholesterol mmol l-1) | **1.44** | | 0.34 | | **1.50** | | 0.54 | *0.58* | **1.54** | 0.41 | **1.52** | | 0.43 | *0.79* | 0.11 | (-0.29 to 0.14) | | 0.5 |
| LDL cholesterol mmol l-1) | **2.85** | | 0.57 | | **2.90** | | 0.26 | *0.84* | **3.20** | 1.13 | **3.39** | | 1.41 | *0.41* | -0.31 | (-0.47 to 0.75) | | 0.44 |
| Triglycerides (mmol l^-1^) | **1.43** | | 0.53 | | **1.15** | | 0.33 | *0.13* | **2.17** | 1.03 | **2.07** | | 1.25 | *0.53* | -0,21 | (-0.24 to 0.60) | | 0.83 |
| ApoD (ug/l) | **123** | | 19,0 | | **123** | | 28.0 | *0.99* | **115** | 14.8 | **105** | | 8.2 | *0.14* | -10.0 | (-22.2 to -52.8) | | 0.57 |
| Weight (kg) | **85.48** | | 23.11 | | **83.42** | | 23.68 | *0.07* | **88.30** | 12.06 | **88.06** | | 12.57 | *0.37* | -0.96 | (-0.33 to -4.09) | | **0.02** |
| BMI (kg m^-2^) | **30.22** | | 6.03 | | **29.47** | | 6.30 | *0.09* | **29.64** | 4.57 | **29.58** | | 4.71 | *0.43* | -0.34 | (-0.03 to -1.39) | | **0.04** |
| Waist circumference (cm) | **91.83** | | 16.88 | | **91.67** | | 16.79 | *0.94* | **97.20** | 11.30 | **92.50** | | 5.43 | *0.19* | -4.52 | (-3.00 to -7.85) | | 0.29 |
| Fat% | **38.02** | | 8.43 | | **33.13** | | 10.29 | *0.2* | **32.72** | 11.01 | **33.34** | | 11.93 | *0.48* | -0.40 | (-0.99 to -14.9) | | 0.09 |
| Visceral fat area (cm^2^) | **161.6** | | 59.5 | | **141.1** | | 39.4 | *0.09* | **161.9** | 22.65 | **163.5** | | 21.50 | *0.62* | -9.90 | (-3.83 to -42.6) | | **0.02** |
| Daily steps were averaged from the 3 months period. High active subject walked 8539±1942 (mean ±SD) and low ones 3463±981 steps per day. | | | | | | | | | | | | | | | | | | |
| *p for within group changes* | |  | |  | |  | | | | | |  | | | | | | |
| *P for difference in change between groups* | | | | | |  | | | | | |  | | | | | | |
| 12 subjects (3 females in both groups) were studied before and after the intervention | | | | | | | | | | | |  | | | | | | |
