# Supplementary material for: Low level activity thresholds for changes in NMR biomarkers and genes in high risk subjects for Type 2 Diabetes
Source: Sci Rep. 2017 Sep 18;7:11267. doi: 10.1038/s41598-017-09753-6 (PMC5603534; doi:10.1038/s41598-017-09753-6)
Supplement: Supplementary file 3 — Supplementary Table 2 [file 41598_2017_9753_MOESM3_ESM.doc]

**Supplementary Table 2**: Principal component analysis of the metabolome measures. Rotated factor pattern was used and values less than 0.3 were excluded.

**Factor1 Factor2 Factor3 Factor4 Factor5 Factor6 Factor7 Factor8 Factor9 Factor10 Factor11**

**Large VLDL particles 0.96331 . . . . . . . . . .**

**Triglycerides in VLDL 0.95369 . . . . . . . . . .**

**Medium VLDL particles 0.94752 . . . . . . . . . .**

**Serum Triglycerides 0.93018 . . . . . . . . . .**

**Very large VLDL particles 0.92588 . . . . . . . . . .**

**Extremely large VLDL particles 0.88236 . . . . . . . . . .**

**Median Diameter of VLDL 0.85798 . . . . . . . . . .**

**Trigly. in Extremely large VLDL 0.84238 . . 0.33010 . . . . . . .**

**Small VLDL particles 0.81538 0.43904 . . . . . . . . .**

**ω7/ω9 fatty acids 0.81286 0.44119 . . . . . . . . .**

**Monounsaturated fatty acids 0.81178 0.40600 . . . . . . . . .**

**Glycoprotein acyls 0.78750 . . . . . . . . . .**

**Total fatty acids 0.77141 0.53955 . . . . . . . . .**

**Small HDL particles 0.66464 . . . . . . . -0.30555 . .**

**Total Cholesterol in HDL3 0.54417 . 0.32897 . . . . . . . .**

**Total Phosphoglycerides 0.52146 0.42889 . . . . . . . . .**

**Large LDL particles . 0.98872 . . . . . . . . .**

**Total cholesterol in large LDL . 0.98860 . . . . . . . . .**

**Total cholesterol in LDL particles. 0.97127 . . . . . . . . .**

**Total Serum Cholesterol . 0.96905 . . . . . . . . .**

**Total cholesterol in IDL particles. 0.95367 . . . . . . . . .**

**Medium LDL particles . 0.94618 . . . . . . . . .**

**IDL particles . 0.93233 . . . . 0.31979 . . . .**

**Small LDL particles 0.30652 0.87956 . . . . . . . . .**

**Triglycerides in IDL particles 0.35601 0.75729 . . . . 0.37682 . . . .**

**Very small VLDL particles . 0.74465 . . . . 0.55179 . . . .**

**ω6 fatty acids 0.46788 0.72303 . . . . . . . . .**

**Apolipoprotein B / ApolipoA1 0.33317 0.66737 -0.56917 . . . . . . . .**

**Phosphatidylcholine& cholines 0.53277 0.60159 . . . . . . . . .**

**Total cholesterol in HDL part. . . 0.96071 . . . . . . . .**

**Total cholesterol in HDL2 part. . . 0.93110 . . . . . . . .**

**Large HDL particles . . 0.93000 . . . . . . . .**

**Total cholesterol in Large HDL . . 0.89037 . . . . . . . .**

**Mean diameter of HDL particles . . 0.86595 . . . . . . . .**

**Extra large HDL particles . . 0.72213 . . . -0.39781 . . . .**

**Valine . . . 0.87153 . . . . . . .**

**Leucin 0.37773 . . 0.87142 . . . . . . .**

**Tyrosine . . . 0.76243 . . . . . 0.31457 .**

**Isoleucine 0.54006 . . 0.70852 . . . . . . .**

**Phenylalanine . . . 0.70672 . . . . . . .**

**3-hydroxybutrate . . . . 0.86531 . . . . . .**

**Acetoactate . . . 0.36634 0.82459 . . . . . .**

**Glucose . . . . 0.69983 . . . . . -0.31201**

**Acetate . . . . 0.57484 . . . . . 0.33755**

**ω3 fatty acids . . . . . 0.85389 . . . . .**

**Double bonds in fatty acids -0.62918 . . . . 0.65313 . . . . .**

**Methylene groups/double bonds 0.61766 . . . . -0.65560 . . . . .**

**Mean diameter for LDL particles -0.40239 . . . . . 0.85036 . . . .**

**Medium HDL particles 0.45682 . 0.45785 . . . 0.47944 . . . .**

**Lactate . . . . . . . 0.80609 . . .**

**Glycerol . . . . . . . 0.76228 . . .**

**Pyruvate 0.41859 . . . . . . 0.61290 . . .**

**Citrate . . . . 0.31455 . . 0.47894 0.30039 . 0.32173**

**Histidine . . . . . . . . 0.76782 . .**

**Glycine . . . . . . . . 0.74479 . .**

**Glutamine . . . . . . . . 0.38789 0.68226 .**

**Alanine 0.31827 . . . -0.30012 . . 0.47111 . 0.57999 .**

**Urea . . . . . . . . . . 0.76159**

**Creatinine . . . . 0.34351 0.30339 . . . . 0.45061**

**Values less than [0.3] are not printed.**
