# Supplementary material for: Low level activity thresholds for changes in NMR biomarkers and genes in high risk subjects for Type 2 Diabetes
Source: Sci Rep. 2017 Sep 18;7:11267. doi: 10.1038/s41598-017-09753-6 (PMC5603534; doi:10.1038/s41598-017-09753-6)
Supplement: Supplementary file 4 — Supplementary Table 3 [file 41598_2017_9753_MOESM4_ESM.doc]

SupplementaryTable 3: List of the most increased (A) and most downregulated genes (B) of subjects with high physical activity compared to those with low activity

| **ProbeName** | **raw**  **p- value** | **Fold Change in High Activity group** | **Fold Change in Low Activity Group** | **Ratio HIGH/LOW activity** | **Gene**  **Symbol** | | **Gene name** | |
| --- | --- | --- | --- | --- | --- | --- | --- | --- |
|  | 1. **Most upreguated genes** | | | | | | | |
| A_23_P38271 | 0.00385 | 1.562 | 0.567 | 2.754 | **MYH2** | | myosin, heavy chain 2, skeletal muscle, adult | |
| A_23_P363344 | 0.00624 | 1.588 | 0.586 | 2.708 | **TPM1** | | tropomyosin 1 (alpha) | |
| A_23_P74609 | 0.00383 | 1.914 | 0.779 | 2.456 | **G0S2** | | G0/G1switch 2 | |
| A_23_P136777 | 0.00083 | 1.179 | 0.508 | 2.323 | **APOD** | | apolipoprotein D | |
| A_23_P35414 | 0.0048 | 1.141 | 0.661 | 1.726 | **PPP1R3C** | | protein phosphatase 1, regulatory subunit 3C | |
| A_24_P76210 | 0.00313 | 1.149 | 0.682 | 1.685 | **A_24_P76210** | | Unknown | |
| A_23_P344515 | 0.00315 | 1.35 | 0.804 | 1.679 | **C16orf3** | | chromosome 16 open reading frame 3 | |
| A_24_P44462 | 0.0033 | 1.194 | 0.715 | 1.67 | **TPM1** | | tropomyosin 1 (alpha) | |
| A_32_P115518 | 0.00079 | 1.315 | 0.792 | 1.661 | **AI207522** | | HA2878 Human fetal liver cDNA library  Homo sapiens cDNA | |
| A_24_P926849 | 0.00112 | 1.346 | 0.834 | 1.615 | **AJ230821** | | clone PS14C5 | |
| A_32_P78101 | 0.00105 | 1.095 | 0.689 | 1.589 | **IGSF21** | | immunoglobin superfamily, member 21 | |
| A_23_P36985 | 0.00413 | 1.193 | 0.762 | 1.567 | **PCDH8** | | protocadherin 8 | |
| A_24_P319675 | 0.00419 | 1.257 | 0.811 | 1.549 | **RAB10** | | RAB10, member RAS oncogene family | |
| A_23_P144980 | 0.00225 | 1.207 | 0.783 | 1.542 | **PIK3R1** | | phosphoinositide-3-kinase,  regulatory subunit 1 (alpha) | |
| A_24_P522678 | 0.00186 | 1.076 | 0.702 | 1.533 | **AF117899** | | Homo sapiens LDLR-FUT fusion protein (LDLR-FUT) | |
| A_23_P201287 | 0.00737 | 1.248 | 0.814 | 1.533 | **KIF1B** | | kinesin family member 1B | |
| A_23_P121253 | 0.00775 | 1.29 | 0.842 | 1.533 | **TNFSF10** | | tumor necrosis factor (ligand) superfamily,  member 10 | |
| A_24_P579439 | 0.0079 | 1.059 | 0.696 | 1.522 | **AF086790** | | aconitase precursor | |
| A_23_P24004 | 0.00744 | 1.419 | 0.932 | 1.521 | **IFIT2** | | interferon-induced protein with tetratricopeptide  repeats 2 | |
| A_32_P160186 | 0.00369 | 1.165 | 0.768 | 1.517 | **EIF5** | | Eukaryotic translation initiation factor 5 | |
| **B) Most downregulate genes** | | | | | | | |  |
| A_23_P115261 | 0.00221 | 0.977 | 1.305 | 0.749 | **AGT** | angiotensinogen (serpin peptidase inhibitor,  clade A, member 8) | |  |
| A_32_P96807 | 0.00294 | 0.871 | 1.169 | 0.745 | **RC3H1** | ring finger and CCCH-type domains 1 | |  |
| A_23_P154585 | 0.00538 | 1.038 | 1.393 | 0.745 | **SNX21** | sorting nexin family member 21 | |  |
| A_23_P433753 | 0.00589 | 0.933 | 1.264 | 0.738 | **PRKAR1A** | protein kinase, cAMP-dependent, regulatory,  type I, alpha (tissue specific extinguisher 1) | |  |
| A_32_P62863 | 0.00099 | 0.86 | 1.183 | 0.727 | **SCHIP1** | schwannomin interacting protein 1 | |  |
| A_23_P132468 | 0.00574 | 0.91 | 1.251 | 0.727 | **SLC4A7** | solute carrier family 4, sodium bicarbonate cotransporter,  member 7 | |  |
| A_23_P132644 | 0.00813 | 1.016 | 1.397 | 0.727 | **NCEH1** | neutral cholesterol ester hydrolase 1 | |  |
| A_23_P131202 | 0.00242 | 0.831 | 1.175 | 0.707 | **HES6** | hairy and enhancer of split 6 (Drosophila) | |  |
| A_23_P47704 | 0.00786 | 0.842 | 1.262 | 0.667 | **UCP2** | uncoupling protein 2 (mitochondrial,  proton carrier) | |  |
| A_24_P67364 | 0.0062 | 0.87 | 1.318 | 0.66 | **TPM3** | tropomyosin 3 | |  |
| A_24_P131646 | 0.00173 | 0.885 | 1.347 | 0.657 | **MYL3** | myosin, light chain 3, alkali; ventricular,  skeletal, slow | |  |
| A_23_P55846 | 0.00654 | 0.885 | 1.348 | 0.656 | **LOC147804** | tropomyosin 3 pseudogene | |  |
| A_32_P80255 | 0.00327 | 0.851 | 1.308 | 0.65 | **DDX6** | DEAD (Asp-Glu-Ala-Asp) box helicase 6 | |  |
| A_24_P249253 | 0.00941 | 0.899 | 1.422 | 0.632 | **LMOD2** | leiomodin 2 (cardiac) | |  |
| A_23_P51565 | 0.00454 | 0.865 | 1.402 | 0.617 | **TNNI1** | troponin I type 1 (skeletal, slow) | |  |
| A_23_P155638 | 0.0015 | 0.862 | 1.407 | 0.613 | **MYL3** | myosin, light chain 3, alkali; ventricular,  skeletal, slow | |  |
| A_23_P88849 | 0.00913 | 0.743 | 1.368 | 0.543 | **RRAD** | Ras-related associated with diabetes | |  |
| A_23_P88404 | 0.003 | 0.997 | 1.888 | 0.528 | **TGFB3** | transforming growth factor, beta 3 | |  |
